# Periodontal Treatment by Dental Undergraduate Students: Assessment of the Patient’s Oral Quality of Life – A Prospective Pilot Study

**DOI:** 10.3290/j.ohpd.b1453071

**Published:** 2021-06-01

**Authors:** Maria-Paloma Alvarez-Azaustre, Manuel Bravo, Antonio Magan-Fernandez, Alberto Rodriguez-Archilla, Carmen Llena, Francisco Mesa

**Affiliations:** a Adjunct Professor, Department of Dentistry, Faculty of Biomedical and Health Sciences, Universidad Europea de Valencia, Valencia, Spain. Hypothesis, experimental design, performed the experiments, wrote and approved the manuscript.; b Full Professor, Department of Preventive and Community Dentistry, School of Dentistry, Campus Universitario de Cartuja, Granada, Spain. Experimental design, statistical analysis and approved the manuscript.; c Postdoctoral Researcher, Department of Periodontology, School of Dentistry, Campus Universitario de Cartuja, University of Granada, Granada, Spain. Proofread, wrote and approved the manuscript.; d Associate Professor, Department of Oral Medicine, School of Dentistry, Campus Universitario de Cartuja, University of Granada, Granada, Spain. Performed experiments, read and approved the manuscript.; e Associate Professor, Department of Stomatology, University of Valencia, Valencia, Spain. Hypothesis, Proofread, wrote and approved the manuscript.; f Full Professor, Department of Periodontology, School of Dentistry, Campus Universitario de Cartuja, University of Granada, Granada, Spain. Conception, hypothesis and design, project supervision, wrote and approved the manuscript.

**Keywords:** dental, oral health, periodontal debridement, periodontitis, students, quality of life

## Abstract

**Purpose:**

To assess the impact of nonsurgical periodontal treatment, performed by undergraduate dental students, on oral health-related quality of life of patients with periodontitis.

**Materials and Methods:**

An observational, prospective, single-arm cohort study with pre-post test involving 31 undergraduate dental students was performed. A complete periodontal examination was performed before and after receiving nonsurgical periodontal treatment. The main independent clinical variables assessed were the degree of periodontal inflammation and the number of teeth with periodontitis. Oral health-related quality of life was assessed before and after treatment through the Oral Impacts on Daily Performances (OIDP) questionnaire. The association between the extent of periodontal treatment (measured as number of treated teeth) and final OIDP score was assessed, adjusting for age, sex, and baseline OIDP, in a multiple regression model.

**Results:**

Thirty-four patients were enrolled and treated by the undergraduate students. The mean OIDP value (global absolute score), representing the severity and frequency of the impacts, decreased from 26.2 to 12 after treatment. The mean percentage of impact, representing the number dimensions affected by oral health (global percent score), was reduced from 13% to 6%. However, no association between the number of treated teeth and post-treatment OIDP score was observed after adjusting for age, sex, and baseline OIDP score.

**Conclusion:**

Nonsurgical periodontal treatment performed by undergraduate dental students improved the oral health-related quality of life of periodontal patients, although no statistically significant association was found between the extent of periodontal treatment and the final OIDP score.

Diagnosis, instructions in oral hygiene and nonsurgical treatment with debridement of the surface of the dental root are periodontal competencies and skills that the undergraduate dental student must acquire.^15^ In a recent study through self-administered questionnaires performed in dental students from the USA, 63.1% of them judged the periodontal care that they provided to their patients as inadequate.^4^ Authors have described several factors to explain these results, such as treatment provided by multiple students over time, supervision by a single student, academic requirements that limit clinical time and prevents the re-evaluation of the patient, and non-compliance with the appointments by the patient to complete the treatment. Albaraki et al^3^ showed that only 4.5% of female patients and 3% of male patients attended the first periodontal re-evaluation by undergraduate students from Riyadh Elm University in Riyadh, Saudi Arabia.

Knowing the opinion of the patient would be an important aspect of the student-patient relationship, since the assessment of nonsurgical periodontal treatment by the patient could be an important part of the learning procedure of the undergraduate student. This could be performed by assessing the satisfaction and/or oral health-related quality of life (OHRQoL) of the patients, through the implementation of a questionnaire before and after treatment. To our knowledge, there is no scientific evidence on this topic.

The Oral Health Status and Oral Impacts on Daily Performances (OIDP) survey for adults was developed and validated in English by Adulyanon and Sheiham,^1^ and is focused on measuring severe oral impairments affecting a person’s ability to perform daily activities. It includes 8 daily-life aspects, such as eating and enjoying food, speaking and pronouncing well, difficulty brushing teeth or using mouthrinse, loss of emotional stability (angry or irritated) due to an oral cause, difficulty resting or sleeping well, embarrassed by the appearance of one’s teeth, working and contact with people. The OIDP index is a relevant tool to assess oral health problems in a specific sociocultural community,^18^ and was validated for adults in Spain by Montero et al in 2008.^11^

The objective of our study was to assess the impact on OHRQoL, measured by OIDP index, of nonsurgical periodontal treatment of patients with periodontitis treated by undergraduate dental students.

## Materials and Methods

### Study Design

The study protocol was approved by the Ethics Committee of Human Research of the University of Granada (Ref. 1138/CEIH/2020). An observational, prospective, single-arm cohort, pre-post study was performed, involving undergraduate dental students from the School of Dentistry of the University of Granada (Spain), in the first semester of the academic year 2019-2020. All patients had been referred to the Periodontology Service of the School of Dentistry and underwent nonsurgical periodontal treatment.

The inclusion criteria were individuals age >18 years with a diagnosis of periodontitis according to the following criteria: presence of at least 2 sites with pocket probing depth (PPD) ≥ 4 mm at 2 different teeth, clinical attachment loss (CAL) ≥ 3 mm, with bleeding on probing (BOP) and bone loss ≥ 2 mm confirmed by orthopantomograms.^14^ Individuals were excluded if they: had undergone antibiotic and/or anti-inflammatory therapy 3 months before the study, had a previous history of periodontal therapy in the last year, had ischemic cardiopathy, were taking anticoagulants, were hepatitis or HIV-positive patients, or had a diagnosis of any psychological disorder that could interfere in the comprehension of the questionnaire. The number of teeth present was not considered as an exclusion criterion. Written informed consent was obtained from all participants. This manuscript was prepared according to the Strengthening the Reporting of Observational Studies in Epidemiology (STROBE) guidelines.^19^

### Sample Size

This pilot study was designed to examine the association between scaling and root planing (no. of treated teeth) and OIDP after treatment. We decided on a sample size of at least 25 patients in order to detect a large effect size (r = 0.5, i.e. the association is measured by correlation) according to Cohen,^5^ with alpha = 0.05 and power = 0.8. Finally, 34 patients were enrolled in the study.

### Periodontal Examination and Treatment

All diagnostic exams and treatments were performed by undergraduate students. A complete periodontal examination was performed on each patient, entering the following periodontal parameters in a periodontal chart: PPD, CAL, BOP,^2^ PI (plaque index),^13^ gingival recession and number of periodontally affected teeth. The exam was performed using a PCP-UNC 15 (Hu-Friedy; Chicago, IL, USA) manual periodontal probe. Inter-examiner calibration was not performed among the students, but all of them had undergone previous training in periodontal probing.

All patients received non-surgical periodontal treatment, which consisted of oral hygiene instructions, supragingival plaque control and subgingival scaling and root planning (SRP). At the first appointment, clinical examination and ultrasonic supragingival calculus removal were performed (DTE, Woodpecker G1 tips, mod. HW-3H; Guilin, China). The second appointment was scheduled for the following week, and dental students performed SRP per quadrant under local anesthesia, using manual periodontal Gracey curettes 5/6, 7/8, 11/12, 13/14 (Hu-Friedy) to eliminate calculus and subgingival biofilm. Four to 6 weeks after treatment, maintenance appointments were scheduled for a periodontal examination and reassessment of initial periodontal parameters, all performed by the same student.

### OIDP Questionnaire

The OIDP questionnaire, validated for adults in Spain by Montero et al,^11^ was administered before treatment and at the appointment 4 to 6 weeks later. The survey was interview-administered by each student to her/his corresponding patient. All students received previous training in OHRQoL and specific characteristics, usage and interpretation of the OIDP survey. The questionnaire consisted of 8 structured questions posed to the patient, regarding 8 dimensions or activities of daily life that could be affected by problems in the mouth (eating, speaking, cleaning teeth, working, social relations, sleeping/relaxing, smiling and emotional status). The total OIDP score of each patient was obtained by adding up the individual values of all dimensions. The maximum possible total OIDP score was 200. Percentage of impact was obtained by the formula: (total score x 100)/200.

All questionnaire data were collected and entered, hand-written, by the dental undergraduate students, and then converted to a spreadsheet database in order to proceed with statistical analysis.

### Statistical Analysis

Statistical analysis was performed using SPSS 20 (IBM; Armonk, NY, USA). To assess the effect of periodontal treatment, the pre-post OIDP comparison was not considered to be the most adequate approach, due to the absence of a control group and also to the fact that the questionnaire was administered by dental students who were supervised. Moreover, a student-patient bond is formed that may alter (improve) the answers while administering the post-treatment OIDP questionnaire. Therefore we used multiple linear regression to analyse the possible association between the extent of periodontal treatment (measured as number of treated periodontal teeth) and final OIDP score, adjusted for age, sex, and baseline OIDP score. If periodontal treatment is the cause of the improvement in OHRQoL, a dose-response relationship would be expected (between number of treated teeth and final OIDP score).

## Results

The study included 34 patients – 18 men (52.9%) and 16 women (47.1%) – with a mean age of 52.6 ± 11.1 (mean ± SD). Age groups and baseline values of the two independent clinical variables included in the study (BOP and number of SRP treated teeth) are described in Table 1.

**Table 1:** Patient’s characteristics at baseline (n = 34)

Variable	n (%)
**Age (years)**	
25–49 50–59 60–71 Mean ± SD	10 (29.4) 12 (35.3) 12 (35.3) 52.6 ± 11.1
**Sex**	
Male Female	18 (52.9) 16 (47.1)
**BOP (%)**	
4–33 34–66 67–87 Mean ± SD	7 (20.6) 17 (50.0) 10 (29.4) 53 ± 24
**SRP (no. of treated teeth)**	
5–14 15–19 20–28 Mean ± SD	12 (35.3) 11 (32.4) 11 (32.4) 16.8 ± 5.3

BOP: bleeding on probing; SRP: scaling and root planing.

Table 2 describes the OIDP score both at baseline and after periodontal treatment. The percentage impact at baseline (referred to the previous 6 months) was 13%, compared to 6% after treatment (referred to the previous 4–6 weeks). In addition, Table 2 shows that the prevalence of impact, referred to the number of affected dimensions, was 2.35 ± 1.77 at baseline and 1.35 ± 1.59 after treatment. Therefore, a greater number of OIDP dimensions were affected at baseline compared to the post-treatment examination. The global absolute score – the mean OIDP score – changed from 26.2 ± 35.6 to 12 ± 31.2, and the global percent score – percentage of the impact – from 13 ± 18% to 6 ± 16%. Therefore, an improvement in OHRQoL was observed in the patients. Considering dimensions of daily life, the most affected ones before treatment in order of relevance were toothbrushing, eating and smiling. After treatment, the most affected ones were smiling, eating, talking and toothbrushing.

**Table 2:** OIDP score (mean±SD) of patients in this study (n = 34) †

Variable	Baseline ‡	After SRP §
No. of Impacts ¶	2.35 ± 1.77	1.35 ± 1.59
Global Absolute score ††	26.2 ± 35.6	12 ± 31.2
Global Percent Score (%) f	13 ± 18	6 ± 16
Dimension percent score‡‡		
Eating Speaking Oral Hygiene Sleeping Emotional state Smiling Occupation Social	20 ± 27 6 ± 22 21 ± 28 6 ± 18 8 ± 21 20 ± 36 4 ± 18 17 ± 34	9 ± 21 6 ± 22 6 ± 17 0 ± 1 3 ± 17 14 ± 31 3 ± 17 7 ± 24

†: Please note that we do not compare pre-post values, since they are based on different recovery periods. ‡: based on the six-month period, before scaling and root planing. §: based on the last 4 to 6-week period. ¶: the sum of impacts with values >0, range 0-8. ††: the sum across 8 dimensions of frequency x severity (see methods), range 0-200. ‡‡: absolute score/200 for the total OIDP and absolute score/25 for each dimension.

There was no statistically significant association measured by Pearson’s correlation analysis between BOP or number of treated teeth (which is a proxy variable for the extent of periodontal disease) with OIDP, measured as number of impacts, or absolute or percent scores (results not shown).

Table 3 shows the association between the number of treated teeth and OIDP score post-treatment, adjusted for age, sex and baseline OIDP values. No statistically significant association was found between the number of teeth treated and the improvement in OHRQoL, although the coefficient of determination (R^2^) of all models is slightly elevated; it is derived from the association with baseline OIDP values. Although clinical evaluation is not the purpose of this study, it should be noted that the study was supervised by four professors, the periodontal chart filled out post-treatment showed that periodontal disease was controlled, and all patients entered supportive periodontal therapy (results not shown).

**Table 3:** Multiple linear regression models of factors associated with OIDP † after SRP (n = 34)

Dependent variable OIDP	R^2^	Factors (β ± SE)			
		Number of treated teeth	Age	Female	Baseline OIDP value ‡
No. of Impacts	0.44	-0.04 ± 0.04	0.03 ± 0.02	0.18 ± 0.45	0.57 ± 0.13*
Percent scores					
Global	0.82	-0.21 ± 0.24	0.13 ± 0.11	-0.69 ± 2.49	0.78 ± 0.07*
Eating	0.61	-0.26 ± 0.45	0.18 ± 0.21	-7.91 ± 4.70	0.57 ± 0.09*
Speaking	0.98	0.02 ± 0.12	0.03 ± 0.06	1.19 ± 1.28	1.00 ± 0.03*
Oral Hygiene	0.43	-0.80 ± 0.46	0.14 ± 0.22	-6.69 ± 4.81	0.37 ± 0.09*
Sleeping	0.09	-0.06 ± 0.04	0.00 ± 0.02	0.22 ± 0.41	0.01 ± 0.01
Emotional state	0.65	0.00 ± 0.36	0.14 ± 0.17	-3.87 ± 3.73	0.64 ± 0.09*
Smiling	0.80	0.08 ± 0.50	0.12 ± 0.23	5.48 ± 4.99	0.75 ± 0.07*
Occupation	0.91	-0.09 ± 0.18	0.12 ± 0.08	0.94 ± 1.89	0.92 ± 0.05*
Social	0.59	0.66 ± 0.54	0.32 ± 0.26	5.70 ± 5.84	0.51 ± 0.09*

* p < 0.05; † based on the last-month period, after scaling and root planing; ‡ based on the six-month period, before scaling and root planing.

## Discussion

Periodontal treatment performed by undergraduate dental students was not associated with an improvement in OHRQoL, as assessed by the OIDP score. Although there was an improvement in the OIDP parameters after periodontal treatment (Table 2), this improvement was not due to treatment, as demonstrated by the lack of a statistically significant association between the number of teeth treated and final OIDP.

OIDP surveys, which have been widely tested in different contexts, have been validated for the Spanish adult population,^11^ as well as for children and adolescents.^6^ Our results indicate that it is not a sensitive approach to evaluate periodontal status both at baseline (Pearson’s correlation, results not shown) and after periodontal treatment. Multivariate linear regression analysis showed that there was no association between the number of treated teeth, a variable of the intensity of the therapy, and the improvement in OHRQoL in the 8 dimensions evaluated by the OIDP survey. There could be several reasons for this result: the presence of an information bias, in which the patients did not want to give negative answers to the students at the post-treatment revision, when asked about the improvement in the different aspects; the different time frame of the two surveys, the first one covering the previous 6 months and the second one the previous 4-6 weeks; the small sample size, where the periodontal condition is not correctly perceived by the patient and shown with this type of survey.

In the present study, the OIDP questionnaire was administered as a personal interview, in which each student interviewed her/his patient. The students previously received an explanatory session of the components and terms of the questionnaire and were trained by administering it to each other. This procedure allowed students to learn this methodology of studies on OHRQoL, and ensured that all the questionnaires were correctly performed, avoiding any loss to follow-up (in our study, all patients completed both surveys). However, it could be possible that the way each patient answered might have been influenced by how the student asked the questions.

Segura et al^17^ reviewed the most used instruments to assess the impact of periodontitis on OHRQoL, and found that the most widely used questionnaire was the Oral Health Impact Profile (OHIP-14), followed by others, among which OIDP is included. The two questionnaires are similar. The former uses a Likert-type scale to measure the negative effects on the performance on daily activities in the last 12 months in seven dimensions. These dimensions are: functional limitation, physical pain, psychological distress, physical disability, psychological disability, social disability, handicaps. The OIDP also uses a Likert-type scale to measure the negative impact on basic daily activities during the last six months. These 8 dimensions are: eating, speaking, hygiene, occupational activities, social relations, sleeping-relaxing, smiling, and emotional state. Both instruments have good psychometric properties and their strengths could be complementary in assessing the impact of oral health on quality of life.^12^ In the present study, the OIDP was chosen, as it was validated for Spain by members of our group.

A recent systematic review^10^ aimed to verify whether oral conditions (tooth loss, periodontal disease, dental caries) were negatively associated with health-related quality of life (HRQoL) in adults. Four out of seven studies reported that periodontal disease impairs HRQoL, and 1 study showed that periodontal disease is positively associated with HRQoL. The authors concluded that mixed and inconclusive findings were observed for the association between periodontal disease and HRQoL.^10^ The OIDP score has been used to assess quality of life in relation to periodontal health in several studies. Wandera et al^20^ used OIDP to assess the impact on periodontal status, determined by the periodontal community index, in pregnant women. They concluded that OIDP showed discriminative validity in identifying women with clinical evidence of tooth loss, but was less convincing in identifying women with clinically defined periodontal disease.

Using the OIDP questionnaire, Santuchi et al^16^ compared the effect on the OHRQoL of two protocols of non-surgical periodontal treatment: SRP by quadrant and Full mouth Disinfection (FMD) in one single session. Both protocols showed an improvement in OHRQoL, with no differences between treatment approaches.^16^ The students at our dental school followed the SRP modality by quadrants for non-surgical periodontal treatment, since scientific evidence showed that the FMD approach does not provide any additional benefit.^9^ Costa et al^7^ recently evaluated the effects of compliance during periodontal supportive therapy in the OIDP scores, and showed that patients who are compliant presented lower OIDP scores compared to the irregular compliers. In 2011, the same group published higher scores of neuroticism and conscientiousness (R^2^ = 68%; p < 0.001) associated with higher OIDP scores among periodontal-maintenance-compliant patients.^8^

To our knowledge, this study shows for the first time that dental students performing periodontal treatment, as part of their education, can be clinically effective, although its impact on the OHRQoL of these patients could not be detected by OIDP scores. This is supported by both correlations of the OIDP scores with the two initial clinical variables at baseline, as well as the correlation between the OIDP results after treatment and the number of teeth that received periodontal treatment. The absence of a trend or gradient with any of the OIDP dimensions in the linear regression model also support this finding.

Limitations of this pilot study are mainly the limited group of patients enrolled and analysed, as well as the absence of a control group. For ethical reasons, a control group could have been enrolled and these patients could have been treated at another time, but academic requirements such as limited clinical time, did not allow this design. The fact that all the surveys were not performed by the same interviewer is another inherent limitation of the university environment in which the study was performed.

## Conclusion

Nonsurgical periodontal treatment performed by undergraduate dental students revealed an improvement in OHRQoL of patients, although no statistically significant association between the extent of periodontal treatment and final OIDP score was shown.

## Oral Impact On Daily Performances (Oidp) Questionnaire For Adults, Validated In Spain

Please answer the following questions, selecting the correct answer for each of the 8 sections:

1.In the last 6 months, have you experienced oral, tooth- or prosthesis-related problems that have caused difficulties in EATING?

Yes

No

If the answer was ‘Yes’, in what measure has this problem affected your life?

No effect (0)

A very minor effect (1)

A fairly minor effect (2)

A moderate effect (3)

A fairly severe effect (4)

A very severe effect (5)

If the problem was present during the whole 6-month period, how often was it?

Less than once a month (1)

About 1-2 times a month (2)

About 1-2 times a week (3)

About 3-4 times a week (4)

Every day or nearly every day (5)

If the problem was present during a part of the whole 6-month period, how long did it last?

5 days or fewer (1)

More than 5 days, up to a month (2)

More than 1, up to 2 months (3)

More than 2, up to 3 months (4)

More than 3 months (5)

Please select the reasons you think caused you problems in EATING in the last 6 months:

Toothache

Dental sensitivity

Carious lesions

Dental fractures

Tooth colour change

Size or form-related problems

Malpositioned teeth

Dental mobility

Dry mouth

Gum bleeding

Gum swelling

Calculus

Oral wounds

Oral malodour

Lump in the face

Loss of permanent teeth

Badly fitting prosthesis

Orthodontic appliances

Difficulty in opening mouth

2. In the last 6 months, have you experienced oral, tooth- or prosthesis-related problems that have caused difficulties in SPEAKING?

Yes

No

If the answer was “Yes”, In what measure has this problem affected your life?

No effect (0)

A very minor effect (1)

A fairly minor effect (2)

A moderate effect (3)

A fairly severe effect (4)

A very severe effect (5)

If the problem was present during the whole 6-month period, how often was it?

Less than once a month (1)

About 1-2 times a month (2)

About 1-2 times a week (3)

About 3-4 times a week (4)

Every day or nearly every day (5)

If the problem was present during a part of the whole 6-month period, how long did it last?

5 days or fewer (1)

More than 5 days, up to a month (2)

More than 1, up to 2 months (3)

More than 2, up to 3 months (4)

More than 3 months (5)

Please select the reasons you think caused you problems in SPEAKING in the last 6 months:

Toothache

Dental sensitivity

Carious lesions

Dental fractures

Tooth colour change

Size or form-related problems

Malpositioned teeth

Dental mobility

Dry mouth

Gum bleeding

Gum swelling

Calculus

Oral wounds

Oral malodour

Lump in the face

Loss of permanent teeth

Badly fitting prosthesis

Orthodontic appliances

Difficulty in opening mouth

3. In the last 6 months, have you experienced oral, tooth- or prosthesis-related problems that have caused difficulties in your ORAL HYGIENE?

Yes

No

If the answer was “Yes”, In what measure has this problem affected your life?

No effect (0)

A very minor effect (1)

A fairly minor effect (2)

A moderate effect (3)

A fairly severe effect (4)

A very severe effect (5)

If the problem was present during the whole 6-month period, how often was it?

Less than once a month (1)

About 1-2 times a month (2)

About 1-2 times a week (3)

About 3-4 times a week (4)

Every day or nearly every day (5)

If the problem was present during a part of the whole 6-month period, how long did it last?

5 days or fewer (1)

More than 5 days, up to a month (2)

More than 1, up to 2 months (3)

More than 2, up to 3 months (4)

More than 3 months (5)

Please select the reasons you think caused you problems in ORAL HYGIENE in the last 6 months:

Toothache

Dental sensitivity

Carious lesions

Dental fractures

Tooth colour change

Size or form-related problems

Malpositioned teeth

Dental mobility

Dry mouth

Gum bleeding

Gum swelling

Calculus

Oral wounds

Oral malodour

Lump in the face

Loss of permanent teeth

Badly fitting prosthesis

Orthodontic appliances

Difficulty in opening mouth

4.In the last 6 months, have you experienced oral, tooth- or prosthesis-related problems that have caused difficulties in SLEEPING & RELAXING?

Yes

No

If the answer was “Yes”, In what measure has this problem affected your life?

No effect (0)

A very minor effect (1)

A fairly minor effect (2)

A moderate effect (3)

A fairly severe effect (4)

A very severe effect (5)

If the problem was present the whole 6-month period, how often was it?

Less than once a month (1)

About 1-2 times a month (2)

About 1-2 times a week (3)

About 3-4 times a week (4)

Every day or nearly every day (5)

If the problem was present during a part of the whole 6-month period, how long did it last?

5 days or fewer (1)

More than 5 days, up to a month (2)

More than 1, up to 2 months (3)

More than 2, up to 3 months (4)

More than 3 months (5)

Please select the reasons you think caused you problems in SLEEPING & RELAXING in the last 6 months:

Toothache

Dental sensitivity

Carious lesions

Dental fractures

Tooth colour change

Size or form-related problems

Malpositioned teeth

Dental mobility

Dry mouth

Gum bleeding

Gum swelling

Calculus

Oral wounds

Oral malodour

Lump in the face

Loss of permanent teeth

Badly fitting prosthesis

Orthodontic appliances

Difficulty in opening mouth

5. In the last 6 months, have you experienced oral, tooth- or prosthesis-related problems that have caused difficulties in your EMOTIONAL STATE?

Yes

No

If the answer was “Yes”, In what measure has this problem affected your life?

No effect (0)

A very minor effect (1)

A fairly minor effect (2)

A moderate effect (3)

A fairly severe effect (4)

A very severe effect (5)

If the problem has been present the whole 6-month period, how often was it?

Less than once a month (1)

About 1-2 times a month (2)

About 1-2 times a week (3)

About 3-4 times a week (4)

Every day or nearly every day (5)

If the problem was present during a part of the whole 6-month period, how long did it last?

5 days or fewer (1)

More than 5 days, up to a month (2)

More than 1, up to 2 months (3)

More than 2, up to 3 months (4)

More than 3 months (5)

Please select the reasons you think caused you problems in your EMOTIONAL STATE in the last 6 months:

Toothache

Dental sensitivity

Carious lesions

Dental fractures

Tooth colour change

Size or form-related problems

Malpositioned teeth

Dental mobility

Dry mouth

Gum bleeding

Gum swelling

Calculus

Oral wounds

Oral malodour

Lump in the face

Loss of permanent teeth

Badly fitting prosthesis

Orthodontic appliances

Difficulty in opening mouth

6. In the last 6 months, have you experienced oral, tooth- or prosthesis-related problems that have caused difficulties in SMILING?

Yes

No

If the answer was “Yes”, In what measure has this problem affected your life?

No effect (0)

A very minor effect (1)

A fairly minor effect (2)

A moderate effect (3)

A fairly severe effect (4)

A very severe effect (5)

If the problem has been present the whole 6-month period, how often was it?

Less than once a month (1)

About 1-2 times a month (2)

About 1-2 times a week (3)

About 3-4 times a week (4)

Every day or nearly every day (5)

If the problem was present during a part of the whole 6-month period, how long did it last?

5 days or fewer (1)

More than 5 days, up to a month (2)

More than 1, up to 2 months (3)

More than 2, up to 3 months (4)

More than 3 months (5)

Please select the reasons you think caused you problems in SMILING in the last 6 months:

Toothache

Dental sensitivity

Carious lesions

Dental fractures

Tooth colour change

Size or form-related problems

Malpositioned teeth

Dental mobility

Dry mouth

Gum bleeding

Gum swelling

Calculus

Oral wounds

Oral malodour

Lump in the face

Loss of permanent teeth

Badly fitting prosthesis

Orthodontic appliances

Difficulty in opening mouth

7. In the last 6 months, have you experienced oral, tooth- or prosthesis-related problems that have caused difficulties in your OCCUPATIONAL ACTIVITIES?

Yes

No

If the answer was “Yes”, In what measure has this problem affected your life?

No effect (0)

A very minor effect (1)

A fairly minor effect (2)

A moderate effect (3)

A fairly severe effect (4)

A very severe effect (5)

If the problem has been present the whole 6-month period, how often was it?

Less than once a month (1)

About 1-2 times a month (2)

About 1-2 times a week (3)

About 3-4 times a week (4)

Every day or nearly every day (5)

If the problem was present during a part of the whole 6-month period, how long did it last?

5 days or fewer (1)

More than 5 days, up to a month (2)

More than 1, up to 2 months (3)

More than 2, up to 3 months (4)

More than 3 months (5)

Please select the reasons you think caused you problems in your OCCUPATIONAL ACTIVITIES in the last 6 months:

Toothache

Dental sensitivity

Carious lesions

Dental fractures

Tooth colour change

Size or form-related problems

Malpositioned teeth

Dental mobility

Dry mouth

Gum bleeding

Gum swelling

Calculus

Oral wounds

Oral malodour

Lump in the face

Loss of permanent teeth

Badly fitting prosthesis

Orthodontic appliances

Difficulty in opening mouth

8. In the last 6 months, have you experienced oral, tooth- or prosthesis-related problems that have caused difficulties in your SOCIAL RELATIONS?

Yes

No

If the answer was “Yes”, In what measure has this problem affected your life?

No effect (0)

A very minor effect (1)

A fairly minor effect (2)

A moderate effect (3)

A fairly severe effect (4)

A very severe effect (5)

If the problem has been present the whole 6-month period, how often was it?

Less than once a month (1)

About 1-2 times a month (2)

About 1-2 times a week (3)

About 3-4 times a week (4)

Every day or nearly every day (5)

If the problem was present during a part of the whole 6-month period, how long did it last?

5 days or fewer (1)

More than 5 days, up to a month (2)

More than 1, up to 2 months (3)

More than 2, up to 3 months (4)

More than 3 months (5)

Please select the reasons you think caused you problems in your SOCIAL RELATIONS in the last 6 months:

Toothache

Dental sensitivity

Carious lesions

Dental fractures

Tooth colour change

Size or form-related problems

Malpositioned teeth

Dental mobility

Dry mouth

Gum bleeding

Gum swelling

Calculus

Oral wounds

Oral malodour

Lump in the face

Loss of permanent teeth

Badly fitting prosthesis

Orthodontic appliances

Difficulty in opening mouth
